# A Review on Bradykinin-Related Peptides Isolated from Amphibian Skin Secretion

**DOI:** 10.3390/toxins7030951

**Published:** 2015-03-18

**Authors:** Xinping Xi, Bin Li, Tianbao Chen, Hang Fai Kwok

**Affiliations:** 1 Faculty of Health Sciences, University of Macau, Avenida de Universidade, Taipa, Macau SAR, China; E-Mails: xinpingxi@umac.mo (X.X.); yb47627@umac.mo (B.L.); 2 Natural Drug Discovery Group, School of Pharmacy, Queen’s University of Belfast, Belfast BT9 7BL, Northern Ireland, UK; E-Mail: t.chen@qub.ac.uk

**Keywords:** amphibian, bradykinin-related peptide, biosynthetic kininogen, bradykinin agonist and antagonist

## Abstract

Amphibian skin secretion has great potential for drug discovery and contributes hundreds of bioactive peptides including bradykinin-related peptides (BRPs). More than 50 BRPs have been reported in the last two decades arising from the skin secretion of amphibian species. They belong to the families *Ascaphidae* (1 species), *Bombinatoridae* (3 species), *Hylidae* (9 speices) and *Ranidae* (25 species). This paper presents the diversity of structural characteristics of BRPs with *N*-terminal, *C*-terminal extension and amino acid substitution. The further comparison of cDNA-encoded prepropeptides between the different species and families demonstrated that there are various forms of kininogen precursors to release BRPs and they constitute important evidence in amphibian evolution. The pharmacological activities of isolated BRPs exhibited unclear structure–function relationships, and therefore the scope for drug discovery and development is limited. However, their diversity shows new insights into biotechnological applications and, as a result, comprehensive and systematic studies of the physiological and pharmacological activities of BRPs from amphibian skin secretion are needed in the future.

## 1. Introduction

The skin secretions of amphibians have been widely studied over the past several decades. A plethora of biologically active compounds have been isolated and identified from their granular glands including alkaloids, steroids, bioorganic amines, peptides and proteins [1,2,3]. Bioactive peptides are predominant and remarkable in amphibian skin secretion as a large amount of antimicrobial peptides, physiological active peptides and neurotransmitter like peptides have been discovered [4,5,6]. These peptides are considered to be an important part of their innate immune system, enabling them to have survived predators and infection from microorganisms for millions of years [2].

Historically, bradykinin (BK) and BRPs from amphibian skin were first reported by Erspamer’s group in the 1960s through the 1970s [7,8]. BK is the final product of the kallikrein–kinin system in the mammalian blood system, and is liberated from three types of kininogens: high molecular weight kininogens and low molecular weight kininogens encoded by a single gene, which are found in humans, and T-kininogens found in rats. All three types consist of a heavy chain and light chain, connecting through a disulphide bridge [9,10,11].

Unlike the releasing processes of BK and its large sized precursors in mammalians, amphibian BK demonstrates extreme differences. There is no kallikrein–kinin system in amphibians [12,13]. BK and BRPs are produced from amphibian skin glands as immune defence peptides, in contrast to playing important roles in endogenous hormonal activity in other higher vertebrate groups [12,13,14,15]. BRPs from amphibians are widely studied not only for their diversity of BK homologues which are displayed as *N*-terminal, *C*-terminal extension, insertion and amino acid substitution, but also for their potential in relation to drug development especially in the fields of blood pressure regulation and inflammatory reactions.

Since 1962, Erspamer and his colleagues reported that BRPs exist in amphibian skin extracts [16]. A large array of BRPs have been isolated and identified from many families, including *Ascaphidae* (1 species), *Bombinatoridae* (3 species), *Hylidae* (9 speices) and *Ranidae* (25 species). More than a hundred results are shown if the search term “amphibian and bradykinin” is used through Uniprot Data base (Pubmed access time: 7 December 2014). Whilst this is not a large amount compared to other kinds of skin defence peptides, such as antimicrobial peptides, of which there are nearly 2000 records in the Data base (Pubmed access time: 7 December 2014), BRPs have the largest number of analogues. When compared to BK, a high and varying degree of structural changes occur through four main characteristics: *N*-terminal extension, *C*-terminal extension, segment insertion and amino acid substitutions and the cloned cDNAs illustrated by the phenomenon of single copy or multiple tandem copies of mature peptide in a full-length cDNA sequence.

## 2. The Distribution of BRPs among Amphibian

### 2.1. BRPs from Ascaphidae

*Ascaphidae* is an ancient amphibian family with only one genus, *Ascaphus*. Representatives of this genus are found in North America in a small area off the west coast of Canada and the United States (The IUCN red list access time: 10 January 2015). Conlon and his colleagues reported BK and three BRPs in the species of *Ascaphus truei* [17]. As shown in Table 1, mammalian BK and an analogue with two Val-Asp amino acid extensions at *C*-terminals were isolated. In addition, the skin glands secreted Ala^0^, Pro^1^, Val^2^, Leu^5^-BK and its analogue extended by Val-Val at *C*-terminals. These peptides were isolated through reverse-phase chromatography and identified by Edman degradation alone without identification of their cDNA-encoded precursors. A five amino acid residues sequence (FSPFR) is identical to BK, which accounts for the three BRPs induced myotropic activities on isolated mouse trachea [17]. It has been postulated that these peptides are liberated from multiple duplications of an ancestral gene. However, due to the lack of records of their encoded gene information, their prepropeptide precursors cannot be determined. This suggests that the processing of the release and proteinase cleavage arising from their precursors requires further study.

**Table 1:** Bradykinin-related peptides (BRPs) isolated from skin secretion of *Ascaphidae.* Peptides were aligned to compare to conventional bradykinin. AR-10, AV-12 and RD-11 were named in accordance of first and last amino acid and the number of amino acid residues.

Name	Peptide Sequence	Species
BK	RPPGFSPFR	*Ascaphus truei* [17]
AR-10	APVPLFSPFR	*Ascaphus truei* [17]
AV-12	APVPLFSPFRVV	*Ascaphus truei* [17]
RD-11	RPPGFSPFRVD	*Ascaphus truei* [17]

### 2.2. BRPs from Bombinatoridae

**Figure 1:**
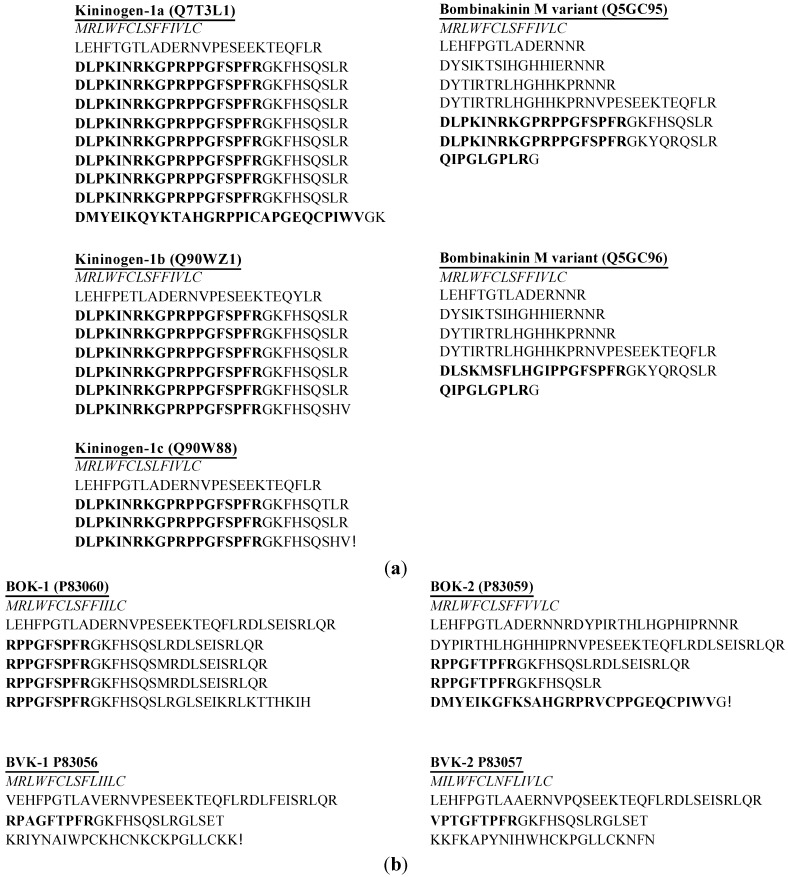
(**a**) cDNA-encoded biosynthetic precursors are identified from skin secretion of *Bombina maxima*; (**b**) cDNA-encoded biosynthetic precursors are identified from skin secretion of *Bombina orientalis* and *Bombina variegate*. BOK and BVK represent preprobradykinin kininogens from *Bombina orientalis* and *Bombina variegate*, respectively. The putative signal peptide sequences are shown in italic typeface and the mature peptide sequences are represented in bold typeface. The access numbers are shown after the name of each precursor.

Only three species have been studied in isolation of BRPs in this family: *Bombina maxima*, *Bombina orientalis* and *Bombina variegata* (Table 2). The most notable peptide group is bombinakinin, including bombinakinin M, an *N*-terminal 10 amino acid residues extended BRP from the skin secretion of *Bombina maxima* [18,19] and bombinakinin O, an *C*-terminal extended BRP, from the skin secretion of *Bombina orientalis* [20]. It was first reported that the cDNA-encoded precursor contained six identical copies of bombinakinin M [18]. However, in subsequent studies, the structures of bombinakinin M prepropeptide precursors have been reported in multiple forms. The prepropeptide precursor containing three identical bombinakinin M copies was reported by Chen *et al.* [19]. One precursor was reported by Lai [21]; it contained eight copies of bombinakinin M and following a bombinakinin-GAP, a 28 amino acid peptide of limited similarity to BK. Another two precursor variants were identified by Lee, one which contains two bombinakinin M replicates and the other contains a single copy (Figure 1a) [22]. It is unclear whether the peptide arising from different cDNA-encoded precursors comes from one single species since different methodologies were used inadvertently [19]. The results from the release of identical mature peptides, shown as the difference of the number of copies in precursors, suggest the possibility of the anomalies occurring in skin secretion due to the variation of gene expression regulation arising for some unknown reason.

Mammalian BK and four analogue peptides with amino acids substitutions have been identified. BRPs from *Bombina orientalis* and *Bombina variegate* were demonstrated to be threonine amino acid residue substitution at position 6, which commonly occurs in other amphibian families such as *Hylidae* and *Ranidae* described below. They demonstrated highly conserved sequences of mature peptides but the cDNA-encoded prepropeptide precursors displayed differential features.

**Table 2:** BRPs isolated from skin secretion of *Bombinatoridae*. Peptides are aligned in order to compare to the conventional BK.

Name	Peptide Sequence	*Species*
BK	RPPGFSPFR	*Bombina orientalis* [23]
Bombinakinin M	DLPKINRKGPRPPGFSPFR	*Bombina maxima* [18,19,21,22]
Bombinakinin O	RPPGFSPFRGKFH	*Bombina orientalis* [20]
Thr^6^-BK	RPPGFTPFR	*Bombina orientalis* [24]
Ala^3^, Thr^6^-BK	RPAGFTPFR	*Bombina variegata* [25]
Val^1^, Thr^3^, Thr^6^-BK	VPTGFTPFR	*Bombina variegata* [25]
pGlu^1^, Ile^2^, Leu^5^, Gly^6^, Leu^8^-BK	QIPGLGPLR	*Bombina maxima* [26]

It has also been reported that the kininogens from *Bombina orientalis* encoded multiple copies, four identical replicates of BK in preprobradykinin-1 (BOK-1) and two identical copies with a variant called DV-28 in preprobradykinin-2 (BOK-2) [23,24]. Compared to the preprobradykinin precursors (BVK-1 and BVK-2) from *Bombina variegate*, four preprobradykinin kininogens demonstrated highly conserved sequences between their signal peptides (Figure 1b) [25]. It is possible that the peptide-encoding gene has evolved into multiple lengths and produced variants of mRNA due to the changes of their living condition.

The pGlu^1^, Ile^2^, Leu^5^, Gly^6^, Leu^8^-BK, namely the kinestatin, was isolated from *Bombina maxima* and the cDNA-encoded prepropeptide has two tandem mature peptide sequences, bombinakinin M and kinestatin. The primary structure revealed little similarity to conventional BK [26]. It antagonizes BK-induced arterial smooth muscle relaxation by targeting the B2 receptor [26]. Similar to Leu^8^-BK which has been reported as a BK receptor antagonist [27], kinestatin also has Leu residue at position 8 and hence demonstrates antagonist activity. Meanwhile, the other site substitutions contribute to structural changes, which may result in the transformation of agonist into antagonist as well.

### 2.3. BRPs from Hylidae

**Figure 2:**
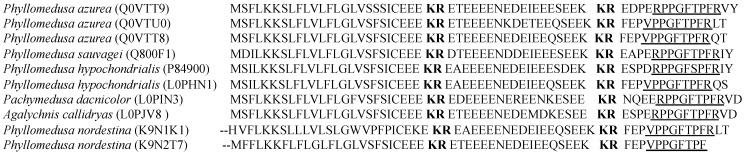
Biosynthetic precursors were identified from skin secretion of the amphibian family, *Hylidae.* The species and their access numbers are shown before the alignments of each precursor. The classic prepropeptide convertase processing sites –KR– are shown in bold typeface. The putative acidic amino acid residues rich peptides were located between two –KR– in each case. The regions with a single underline indicate highly conserved sequences as compared to conventional BK.

*Hylidae* is one of the largest families among amphibians, containing 944 species. The BRPs discovered in this family all have been reported from *Phyllomedusinae*, a sub-family which contains 59 species [28]. The BRPs isolated from *Phyllomediusinae* display only one single copy of mature peptide sequence in their precursors and show identical topological structures, as opposed to the peptides isolated from *Bombinatoridae* and *Ranidae*, which display multiple copies of mature peptide sequence in the precursors (Figure 2) [29,30,31]. One possible reason behind the difference in proprepeptide sequence is because the species in this sub-family spend almost their entire lives on the tree during the spawning and tadpole stage, and this lifestyle helps to maintain secretions over their surface more easily than amphibians living in the water or buried in sand, mud and even fallen leaves. This unique lifestyle possibly reduces the necessity of expressing different defence peptide precursors to adapt to different living conditions.

BRPs aligned here (Table 3) generally demonstrate the diverse BRPs sequences isolated from *Phyllomedusinae* skin secretion. Amino acid residue substitutions mainly occurred at two sites: valine for arginine at position 1 and threonine for serine at position 6. Of interest, some desArg^9^ peptides were isolated from skin secretion of *Hylidae* and *Ranidae.* The presence of desArg^9^ BK and BRPs, the peptides showing a high degree of affinity for the B_1_ receptor, indicates that targeting of the B_1_ receptor might be an important defence strategy against their predators. However, another possibility is that because there are some internal enzymes like ACE in the amphibian body, they may cleave the Arg^9^ from their conventional peptides. This interaction may contribute to the presence of these desArg^9^ peptides in their skin secretion.

**Table 3:** BRPs isolated from skin secretion of *Hylidae*. Peptides are aligned to compare to conventional BK. The proline residues with single underline represent the post-translational modification of hydroxyproline. The tyrosine residues with double underline represent the post-translational modification of *O*-sulfate.

Names	Peptide Sequences	Species
BK	RPPGFSPFR	*Phasmahyla jandaia* [32] *Phyllomedusa azurea* [30] *Phyllomedusa hypochondrialis* [33,34]
desArg^9^-BK	RPPGFSPF	*Phasmahyla jandaia* [32]
Hyp^3^-BK	RPPGFSPFR	*Phyllomedusa azurea* [30]
desArg^9^, Hyp^3^-BK	RPPGFSPF	*Agalychnis callidryas* [35]
Thr^6^-BK	RPPGFTPFR	*Agalychnis callidryas* [31,35] *Pachymedusa dacnicolor* [31] *Phasmahyla jandaia* [32] *Phyllomedusa azurea* [30] *Phyllomedusa hypochondrialis* [31,33,34]
desArg^9^, Thr^6^-BK	RPPGFTPF	*Phasmahyla jandaia* [32] *Phyllomedusa azurea* [30] *Phyllomedusa hypochondrialis* [31,33,34]
Hyp^3^, Thr^6^-BK	RPPGFTPFR	*Agalychnis callidryas* [31] *Pachymedusa dacnicolor* [31]
Thr^6^-BK-Val, Asp	RPPGFTPFRVD	*Pachymedusa dacnicolor* [31] *Agalychnis callidryas* [31]
Hyp^3^, Thr^6^-BK-Val, Asp	RPPGFTPFRVD	*Agalychnis callidryas* [31] *Pachymedusa dacnicolor* [31]
Val^1^, Thr^6^-BK	VPPGFTPFR	*Phyllomedusa azurea* [29,30] *Phyllomedusa hypochondrialis* [31,34] *Phyllomedusa sauvagei* [29]
Val^1^, Hyp^2^, Thr^6^-BK	VPPGFTPFR	*Phyllomedusa azurea* [30]
desArg^9^, Val^1^, Thr^6^-BK	VPPGFTPF	*Phyllomedusa hypochondrialis* [31,34]
Val^1^, Thr^6^-BK-Leu	VPPGFTPFRL	*Phyllomedusa azurea* [30]
Val^1^, Thr^6^-BK-Leu, Thr	VPPGFTPFRLT	*Phyllomedusa azurea* [30]
Glu, Pro-Val^1^-BK-Leu, Thr	EPVPPGFTPFRLT	*Phyllomedusa azurea* [30] *Phyllomedusa nordestina* [36]
Val^1^, Thr^6^-BK-Gln	VPPGFTPFRQ	*Phyllomedusa hypochondrialis* [31,34]
Val^1^, Thr^6^-BK-Gln, Ser	VPPGFTPFRQS	*Phyllomedusa azurea* [30] *Phyllomedusa hypochondrialis* [31,34]
Val^1^, Hyp^2^, Thr^6^-BK-Gln, Ser	VPPGFTPFRQS	*Phyllomedusa azurea* [37,38] *Phyllomedusa hypochondrialis* [31,34]
Val^1^, Hyp^2^, Thr^6^-BK-Gln, Thr	VPPGFTPFRQT	*Phyllomedusa azurea* [30]
Val^1^, Thr^6^-BK-Gln, Asp	VPPGFTPFRVD	*Phyllomedusa hypochondrialis* [31,34]
PK *	RPPGFSPFRIY	*Phasmahyla jandaia* [32] *Phyllomedusa bicolor* [39] *Phyllomedusa hypochondrialis* [33,34] *Phyllomedusa rohdei* [40]
Hyp^3^-PK *	RPPGFSPFRIY	*Agalychnis callidryas* [35] *Phyllomedusa hypochondrialis* [33,34]
Thr^6^-PK *	RPPGFTPFRIY	*Pachymedusa dacnicolor* [41,42] *Phasmahyla jandaia* [32] *Phyllomedusa hypochondrialis* [33,34] *Phyllomedusa sauvagei* [29]
Hyp^3^, Thr^6^-PK *	RPPGFTPFRIY	*Pachymedusa dacnicolor* [41,42] *Phyllomedusa sauvagei* [29]
Thr^6^, Val^10^-PK *	RPPGFTPFRVY	*Phyllomedusa azurea* [30]
Asp, Pro, Glu-Thr^6^, Val^10^-PK *	DPERPPGFTPFRVY	*Phyllomedusa azurea* [30]

* Both *O*-sulfate and non-sulfate forms of phyllokinin identified from the skin secretion in each case.

There are only two isolated BRPs displaying the structural variation of *N*-terminal extensions. They are Glu, Pro-Val^1^-BK-Leu, Thr and Asp, Pro, Glu-Thr^6^, Val^10^-phyllokinin (PK). However, in the reported data of the cDNA encoded peptide precursors from the skin secretion of *Phyllomedusinae* frogs, all BRPs demonstrated this similar *N*-terminal extension of tri or tetra amino acid residues in their prepropeptide precursors and the extended region all followed one typical propeptide convertase processing site, –KR– (Figure 2). In most cases, the extended region contains acidic amino acid residue and they were cleaved to generate the mature peptides. It is speculated that the release of mature peptides might be processed by the cleavage at the –KR– site followed by a specific enzymatic interaction. It was explained as a particular structural feature for producing post-translational modification of these BRPs [29,30], and more research is required.

The unique kind of peptide in the skin secretion of sub-family *Phyllomedusinae* is phyllokinin (PK), which displays two amino acid residues Ile-Tyr extended at *C*-terminals. PK was first reported from the studies of skin of *Phyllomedusa rohdei* with a post-translational modification of tyrosine *O*-sulfation and this peptide was subsequently discovered from other species in this sub-family [29,32,33,35,41,42]. Similar to PK, some of the BRPs were *C*-terminal extended with one or two amino acid residues, which are considered to be more potent than conventional BK because the extended region could inhibit the enzymatic metabolism of degradation of the BRPs, which prolong the ligand-receptor interaction [43]. However, it was reported that the influences of this kind of *C*-terminal extension are different in their pharmacological effect on multiple tissue preparation [34]. Although these analogues have less potency compared to conventional BK, they were believed to make sense in colubrid and crotalid snake bodies because these BRPs resemble the plasma kinin in the kallikrein–kinin system of these predators, and the hypothesis needs to be tested with more target tissue preparations [30].

Post-translational modification of hydroxylation at position 2 or 3 occurred in some BRPs. The pharmacological effect of hydroxyproline modification was reported in the study of comparative effects of multiple BRPs on mammalian smooth muscles [31]. Four BRPs and their hydroxylated analogs revealed differentiated bioactive potency on different isolated smooth muscle tissues, which could be caused by a different metabolism pathway or even a different subtype of receptor [31]. Another special modification occurring in PK is the *O*-sulfated Tyr residue. Both modified and non-modified forms were reported to exhibit BK-agonist activity and the non-modified form was less potent than the *O*-sulfated form [44]. In most reported studies, both forms were isolated from the skin secretion except for a single study on *Agalychnis callidryas*, which may be due to the limited techniques for their research at that moment [35]. Apparently, there is a special sulfatase widely distributed in the *Phyllomedusinae* tree frogs, which contributes to the generation of PK in the skin secretion for the purpose of self-defence. In the process of review, there is a non-uniform nomenclature, which is causing confusion between “PK” and “PK sulfated” in some cases [29,30,34,42]. Considered in the first publication of PK, which is a sulfated form in skin secretion, it should be named the Tyr residue *O*-sulfated form of PK in future studies in order to avoid any misunderstanding.

### 2.4. BRPs from Ranidae

Family *Ranidae* is the most widely distributed amphibian in the world, with exceptions in Antarctica, southern South America and most of Australia [28]. The discovery of BRPs have been reported from the skin secretions of 25 species in this family, and the number of the species is approximately three times more than investigated species in *Hylidae*. They displayed the diversity of structural characteristics not only in their encoded kininogen precursors but also in their mature peptides.

**Figure 3:**
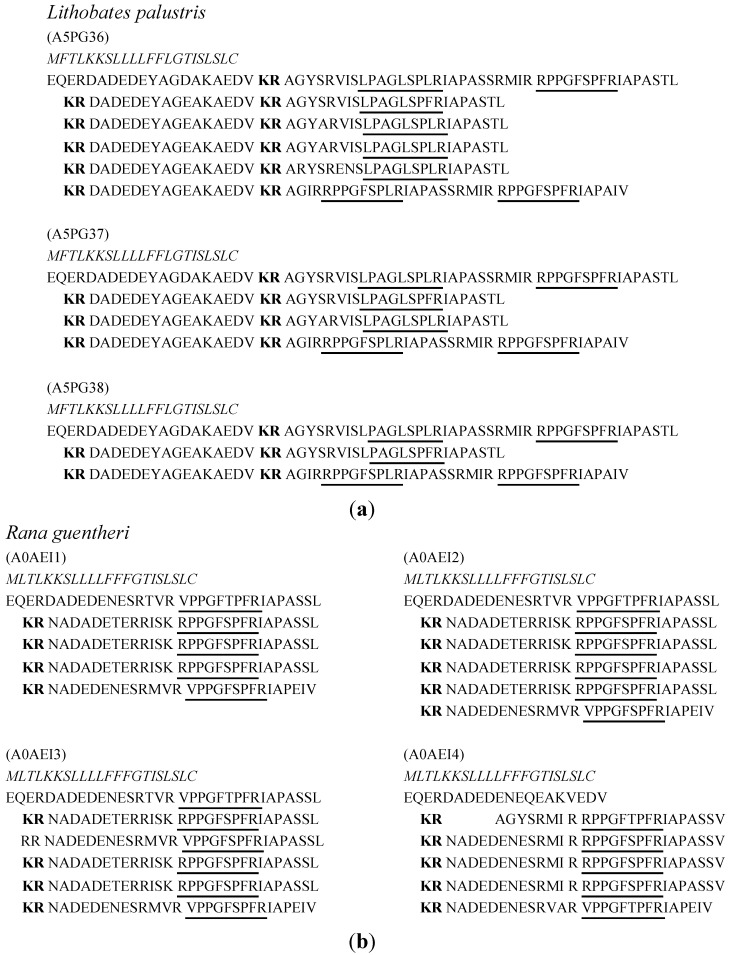

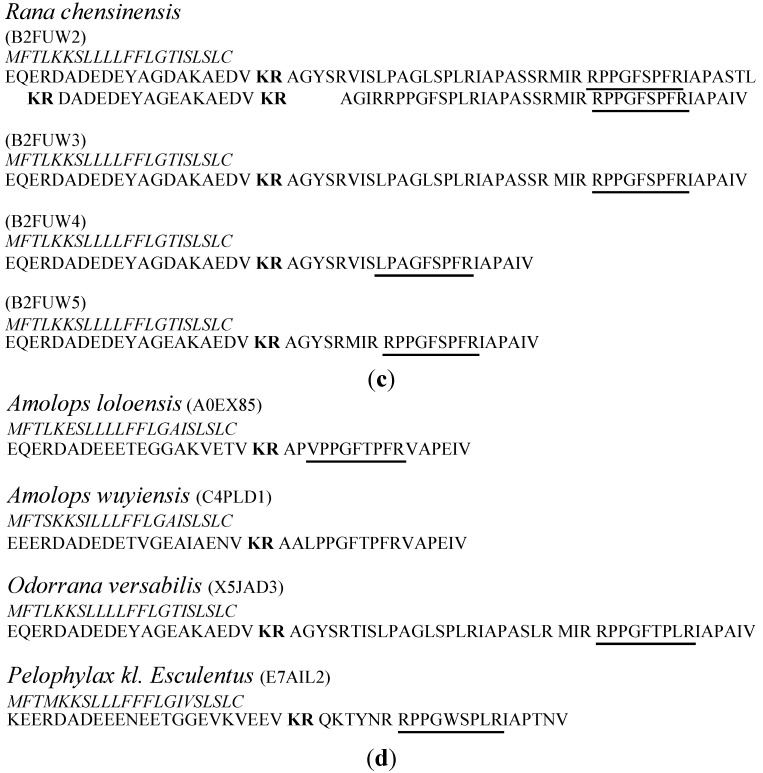
Biosynthetic skin prepropeptides are identified from skin secretion of multiple species from *Ranidae.* (**a**) Three BRP precursors are identified from *Lithobates palustris*; (**b**) Four skin prepropeptides are identified from *Rana guentheri*; (**c**) Four prepropeptides were discovered from *Rana chensinensis*; (**d**) The skin prepropeptides containing single copies were discovered in skin secretion of multiple *Ranidae* species. The access numbers of the Uniprot database are shown at the head of each precursor. The regions shown in italic typeface are signal peptides. Classic prepropeptide convertase processing sites, –KR–, are shown in bold typeface. The regions with a single underline indicate highly conserved sequences as compared to conventional BK.

Similar to cDNA-encoded proprepeptides discovered from *Bombinatoridae*, the proprepeptides in *Ranidae* skin secretion demonstrated different forms of proprepeptides, including tandem copies containing proprepeptides and a single copy containing proprepeptides as well (Figure 3). However, their precursors demonstrated more significant and complicated patterns when compared to other amphibian families. Firstly, the length of the region between the –KR– (classic prepropeptide convertase processing site) and the mature BRPs was longer than the precursors from the species of *Hylidae*. Secondly, more than one sequence of BRP repeatedly appeared in the proprepeptides in some cases. Thirdly, for BK or some BRP expressed in the same species, there were some proprepeptides containing a different number of copies. Due to lack of data and limited research, it was not clear why the encoded proprepeptides of BRPs in this family were significantly different. This phenomenon was probably due to the wide distribution of studied *Ranidae* species, their different living environments and the different species, which influence the expression changes of BRPs. In a sense, the variety of proprepeptides demonstrated the evidence of evolution among the amphibian.

The BRPs isolated from the skin secretion of *Ranidae* species so far are shown and aligned in Table 4. Compared to the BRPs identified in *Hylidae*, the post-translational modification occurred less frequently in this group. Only two studies reported modified BRP, Hyp^3^-BK, discovered from skin secretions of *Hylarana guentheri* and *Rana temporaria* [45]. However, the forms of amino acid substitutions that occurred in BRPs from *Ranidae* were more diverse than the BRPs in other amphibian families. There are some common substitutions including arginine replaced by valine at position 1 [46], serine replaced by threonine at position 6 and phenylalanine replaced by leucine at position 8, which are similar to the amino acid substitutions of BRPs in *Hylidae* and *Bombinatoridae*. Meanwhile, some unique BRPs such as Asp^6^-BK, Arg^0^, Trp^4^, Leu^8^-BK and Arg^0^, Leu^1^, Thr^6^, Trp^8^-BK were isolated in the skin secretion of *Ranidae* [47].

**Table 4:** BRPs isolated from skin secretion of *Ranidae*. Peptides are aligned to compare with conventional BK. The proline residues with single underline represent modification of hydroxyproline.

Names	Peptide Sequences	Species
BK	RPPGFSPFR	*Hyla arborea schelkownikowi* [48] *Lithobates pipiens* [49] *Odorrana grahami* [50] *Rana chensinensis* [49] *Hylarana guentheri* [46] *Rana tagoi okiensis* [51] *Pelophylax ridibundus* [47] *Rana muscosa* [52] *Rana temporaria* [45]
desArg^9^-BK	RPPGFSPF	*Rana temporaria* [45,53]
desArg^1^-BK	PPGFSPFR	*Hylarana guentheri* [46]
Hyp^3^-BK	RPPGFSPFR	*Hylarana guentheri* [46] *Rana temporaria* [45]
Thr^6^-BK	RPPGFTPFR	*Hylarana guentheri* [46] *Rana tagoi okiensis* [51] *Pelophylax ridibundus* [47]
Thr^6^, Leu^8^-BK	RPPGFTPLR	*Odorrana versabilis* [54]
Leu^5^, Thr^6^-BK	RPPGLTPFR	*Odorrana grahami* [50]
Asp^6^-BK	RPPGFDPFR	*Pelophylax ridibundus* [47]
Val^1^-BK	VPPGFSPFR	*Hylarana guentheri* [46]
Val^1^, Thr^6^-BK	VPPGFTPFR	*Lithobates pipiens* [49] *Hylarana guentheri* [46]
BK-Ile	RPPGFSPFRI	*Pelophylax ridibundus* [47]
BK-Ile, Ala	RPPGFSPFRIA	*Pelophylax ridibundus* [47]
BK-IAPAS	RPPGFSPFRIAPAS	*Pelophylax ridibundus* [47] *Lithobates pipiens* [49]
BK-IAPASIL	RPPGFSPFRIAPASIL	*Rana temporaria* [45,53]
Thr^6^-BK-Ile, Ala	RPPGFTPFRIA	*Pelophylax ridibundus* [47]
Thr^6^-BK-IAPAS	RPPGFTPFRIAPAS	*Lithobates pipiens* [49]
BK-VAPAS	RPPGFSPFRVAPAS	*Odorrana schmackeri* [55]
Arg^0^, Trp^5^, Leu^8^-BK	RRPPGWSPLR	*Pelophylaxkl. esculentus* [56]
IR-Leu^8^-BK	IRRPPGFSPLR	*Lithobates palustris* [57,58]
IR-Leu^8^-BK-IA	IRRPPGFSPLRIA	*Lithobates palustris* [59,60]
AGIR-Leu^8^-BK	AGIRRPPGFSPLR	*Lithobates palustris* [59,60]
AGIR-Leu^8^-BK-IA	AGIRRPPGFSPLRIA	*Rana chensinensis* [49] *Lithobates palustris* [59,60]
LLPIVG-BK	LLPIVGRPPGFSPFR	*Rana temporaria* [45]
Arg^0^, Leu^1^, Thr^6^, Trp^8^-BK	RLPPGFTPWR	*Rana sakuraii* [60]
RAA-Leu^1^, Thr^6^-BK	RAALPPGFTPFR	*Amolops wuyiensis* [61]
RVA-Leu^1^, Thr^6^-BK	RVALPPGFTPFR	*Amolops wuyiensis* [61]
RAEA-Val^1^, Thr^6^-BK	RAEAVPPGFTPFR	*Hylarana nigrovittata* [62]
RAP-Val^1^, Thr^6^-BK	RAPVPPGFTPFR	*Amolops loloensis* [59]
Thr^6^-kallidin	KRPPGFTPFR	*Hylarana guentheri* [46]
RLS-Thr^6^-kallidin	RLSKRPPGFTPFR	*Hylarana guentheri* [46]

The names of species *Rana ridibunda*, *Rana palustris* and *Rana nigrovittata* are redirected from *Pelophylax ridibundus*, *Lithobates palustris* and *Hylarana nigrovittata* respectively.

BPRs in this group demonstrated longer extended *C*-terminals and prepropeptides demonstrated two features from most recent reports detailed in Figure 3 [54,56]. Firstly, they are highly conserved through this amphibian family and they identified the regions containing an identical triple amino acids sequence, –IAP–, after Arg^9^. Secondly, the extended *C*-terminal regions are identical within one prepropeptide except the one following the last copy of the putative mature peptide. These extensions are not only displayed in their encoded proprepeptides but also identified from their skin secretion such as BK-IAPASIL, a heptapeptide extension [45]. It was also reported that the *C*-terminal extended BRPs were identified using high resolution mass spectra analysis, along with sequences, which the amino acid residue at *C*-terminals removed subsequently such as RL-16, RS-15, RS-14, RA-13, RP-12, RA-11, RV-10 and BK from the skin secretion of five *Rana* species [53,63]. Apparently, this indicates a complicated pathway for mature peptide release or metabolization. Accordingly, it has raised a question as to what is the right sequence of mature BRPs in the amphibian skin defence strategy.

The structural characteristic of *N*-terminal extended BRPs is much similar to the situation of *C*-terminal extension. There is a highly conserved region in the front of putative mature BRPs in their prepropeptides precursors. In the report of the BRPs isolated from *Rana palustris* (name redirected from *Lithobates palustris*) [57], the *N*-terminal extended sequence, –AGIR– was located between –KR– and the mature peptide sequence. It probably indicates these BRPs were liberated from smaller kininogenin amphibian skin so that both extensions of *C*- and *N*-terminals consist of their kininogen precursor. Meanwhile, a special *N*-terminal extension occurred in this family, by which the putative mature BRPs contain an inserted fragment regionlike sequence, –APV–, first reported in isolated BRPs from *Amolops loloensis* [59], known as amolopkinin, following a report of an insertion of –AEAV– in the BRPs from *Hylarana nigrovittata* [62]. However, the report of insertions inamolopkinin-W1 and W2 from *Amolops wuyiensis* were not mentioned though these two BRPs showed inserted sequence V/A-AL in their sequences [61]. In another respect, the BRPs containing the inserted region could also be regarded as a form of *N*-terminal extension occurring in the BRPs with Val^1^ or Leu^1^ substitution.

## 3. Pharmacological Activities of Isolated BRPs

In 1909, the kallikrein–kinin system (KKS) was discovered by Abelous and Bradier by observing a decrease of blood pressure in humans after the intravenous injection of components extracted from human urine [64]. Since then, this system has been intensively investigated and proven to be an important endogenous and spontaneous metabolic cascade found in many species. The activities of the KKS gravitate around the release of a series of vasoactive kinins, such as BK, that participate in control of blood pressure, inflammatory and cardioprotective processes [65]. Further clinical applications have arisen from recent studies on signalling pathways for their receptors, B1 and B2, such as a promising tumour and inflammatory treatment through the Ras/Raf/MEK/MAPK pathway [66,67,68].

BRPs have a wide distribution among amphibian species and are a component of their innate defence system. They demonstrate a highly conserved sequence as compared to mammalian BK with multiple structural modifications, which indicates unique characteristics for targeting the plasma system or smooth muscle receptors of their predators, such as birds and snakes. Although more than 50 different BRPs have been isolated from amphibian skin secretion, nearly half of them report pharmacological activity on isolated mammalian smooth muscle preparations (Table 5). Most of these illustrated agonized activity on BK receptors with a variety of potency but some appeared to be antagonists. In relation to the conventional BK sequence, Arg^1^, Pro^2^, Gly^4^, Phe^5^, Pro^7^, Phe^8^ and Arg^9^ are necessary components for its biological activity; thus, amino acid substitution and modification could enhance or reduce the potency and even change an agonist into antagonist [69].

Val^1^ and Thr^6^ were the most common amino acid substitution of BRPs from amphibian skin secretion. In comparison to BK, Val^1^-BK had a less potent effect on the rat ileum and demonstrated barely any activity on arteries [46]. The Thr^6^-BK was reported to demonstrate an equivalent effect to BK on the isolated rat artery but having two-fold maximum contraction with higher EC_50_ on rat ileum [23]. As expected, their analogues which contain post-translational modifications including *C*-terminal extension and hydroxylation at position 2 or 3 demonstrated less potent pharmacological effects although their structure–activity relationship has not been explained clearly [25,31].

There were some BRPs from amphibian skin secretion which exhibited potent antagonist activity. DesArg^9^, Leu^8^-BK was reported as a B1 receptor antagonist on the rabbit aorta and artery [70]. In reviewed peptides, two BRPs, Arg^0^, Trp^5^, Leu^8^-BK and pGlu^1^, Ile^2^, Leu^5^, Gly^6^, Leu^8^-BK, induced inhibitory activity against BK induced vascular relaxation. The former induced a 70% reduction of relaxation, and the latter inhibited the response of BK almost completely and it has been confirmed that its antagonist activity was mediated through the B2 receptor [26,56]. It was reported that Phe at position 8 of BK is one of the important sites for activating BK receptors, and the substitution of Leu could induce an antagonist activity [71]. However, a report of Thr^6^, Leu^8^-BK displayed an agonist activity, which produced a 336-fold decrease of EC_50_ on rat ileum and a 10-fold decrease of EC_50_ but increased contractions of the uterus as compared with BK [54]. Compared to the antagonists discussed above, Thr^6^, Leu^8^-BK demonstrated a higher similarity to the amino acid sequence of conventional BK, which may remain potent on activating isolated tissues and it also could be explained that rat ileum and uterus have different types and distributions of receptors as compared with smooth artery muscles.

BRPs containing different terminal extensions exhibited different patterns of bioactive activity. RAA-Leu^1^, Thr^6^-BK and RVA-Leu^1^, Thr^6^-BK isolated from *Amolops wuyiensis* were reported as BK antagonists [61]. However, the two highly similar analogues of RAP-Val^1^, Thr^6^-BK from the same genus, *Amolops loloensis* and RAEA-Val^1^, Thr^6^-BK from *Hylarana nigrovittata* exhibited activities of BK agonists on guinea pig ileum [62]. Thereby, this indicates that BRP receptors may have different subtypes in mammalian species, which contribute to remarkable pharmacological effects. Another probable explanation for their structure indicates that the Val^1^ substituted BRP demonstrates a more potent agonistic behaviour than Leu^1^ substitution. In addition, the BPR containing the longest *N*-terminal extension, Bombinakinin M, is a B_2_ receptor selective agonist and it was proven that it caused prolonged signalling [72]. The longer extended region possibly decreased affinity of aminopeptidase binding, which might prolong the interaction time between BRPs and receptors. The *C*-terminal extensions influenced the potency of BRPs as well. When comparing AV-12 with AR-10, *C*-terminals extended region encoded within a –VV dipeptide, resulted in a nearly 30% reduction of BK induced maximal vascular relaxation [17]. However, PK demonstrated that a dipeptide extension as –IY was more potent than BK on the blood pressure of a dog [44].

It is currently not possible to explain the diversity of pharmacological effects of BRPs which have been isolated from amphibian skin secretion. The hypotheses are usually raised through differential ligand-receptor binding pathways caused by changes of BRPs structural conformations including the extensions and amino acid substitutions. One probable reason could be that these changes prevent the peptide degradation by endogenous enzymes, which induce a decrease of metabolic effects [65,73]. Considering that the function of venomous skin secretion is protection and defence, different BRPs may have specific targets on either the plasma systems or intestinal smooth muscle of various animals that are preying on amphibians. This may be true because related BRP analogs have been isolated and identified from mammals, birds, reptiles and fish [15,37,54,56].

**Table 5:** Pharmacological effects of selected BRPs grouped as BK agonist and antagonist.

BRPs	Pharmacological Effect
**Agonist**	
RPPGFTPLR	Contract the rat ileum; increase contraction frequency in the rat uterus [54].
RPPGFTPFR RPPGFTPFR RPPGFTPFRVD RPPGFTPFRVD	Activating mammalian arterial smooth muscle bradykinin receptors; contract rat ileum, bladder and uterine [31,34].
VPPGFTPFR VPPGFTPFR VPPGFTPFRQS VPPGFTPFRQS	Contract the rat ileum and guinea pig ileum preparations [31,34].
RPAGFTPFR VPTGFTPFR	Relax pre-contracted rat arterial, contract rat ileum [25].
RPPGFSPFRIY	Decrease dog blood pressure [44].
RAPVPPGFTPFR RAEAVPPGFTPFR	Contractile effects on isolated guinea pig ileum [59].
DLPKINRKGPRPPGFSPFR	Contract guniea pig ileum; B_2_ receptor selective agonist [72].
**Antagonist**	
RPPGFSPL	B1 receptor antagonist on the rabbit aorta and artery [70].
RRPPGWSPLR	Antagonize the relaxation in rat arterial smooth muscle induced by bradykinin [26,56].
RVALPPGFTPFR RAALPPGFTPFR	Antagonize the contractile effects of bradykinin on isolated rat ileum smooth muscle preparations [61].
RVALPPGFTPLR	B2 receptor antagonist on rat tail artery [74].
QIPGLGPLR	B2 receptor antagonist on the rat artery [26,56].

## 4. Conclusions

Many studies around the world have indicated that amphibians are a virtual goldmine for further discovery of new drugs in relation to new therapeutic applications. Amphibians generate BK or BRPs in their skin secretion instead of releasing them into plasma. Regarding the natural roles of these BRPs, the hypothesis is that they act as skin defence compounds mediated on the BK receptor of their predators. Firstly, the BK or BRPs are abundant in the skin secretions, which were analysed using liquid chromatography systems in the reviewed papers. From the perspective of evolution, it makes sense that the amount of BK or BRPs should contribute to the skin defence strategy. Secondly, some BRPs have been identified in the plasma of the species which prey on amphibians, such as Val^1^, Thr^6^-BK in snakes, Leu^2^, Thr^6^-BK in lizards and Thr^6^, Leu^8^-BK in birds [15]. Meanwhile, homologues were also detected in the skin secretion of amphibians, which could target the specific BK receptor of their predators. It is speculated that sufficient amounts of BRPs could stimulate the gastrointestinal system resulting in vomiting reflex and ejection. Differences in the primary structure of BPRs in the amphibian skin secretion varies along with the plasma kinins in the predator species and differences in the primary structure of the receptors do exist, specifically between phylogenetically distant animals. However, some uncertainties remain. It is not clear how the BRPs affect the predator. Furthermore, considering that a variety of predator species still prey on amphibians, how are these able to overcome the effect of the BRPs secreted by their prey? Zhou *et al.* proposed that these BRPs genes became pseudogenes in amphibians [75]. In this regard, further studies are necessary to investigate the specificity of the interaction mechanism between BRPs and BK receptors in predators, as well as phylogenetic research on the BRPs and related receptors of both predators and the amphibians themselves.

BRPs from amphibian skin secretion are diverse and they provide a natural-selected sequence storehouse for physiological and pharmacological screening. In the reviewed paper, the activity of the studied BRPs was highly variable. Considering they were subjected to smooth muscle assays for biological screening, there was only limited data obtained by the authors by which to deduce the relationship between function and structure. In order to explain the manner through which BRPs interact with BK receptors to induce agonist and antagonist activity, it is essential to perform a more accurate research approach to evaluate the affinity of the ligand–receptor interaction, as well as their specificity, which is dependent on their spatial structural characteristics.

It is not possible to identify significant candidates for clinical use through BRPs from amphibian skin secretion due to the limited amount of high quality data currently available. However, it brings new insights for biotechnological application. Charest-Morin *et al.* fused bombinakinin M to the enhanced green fluorescent protein (EGFP) with a slight change of affinity of bombinakinin M binding to B2 receptor [76]. Due to resistance to peptideases, the *N*-terminal extension between receptor binding region and EGFP becomes a suitable spacer, which is not able to interfere with the conformation for activating B2 receptor. As a potential source, BRPs constitute the innate defence system of amphibians, while providing a diversity of structural characteristics. Comprehensive and systematic studies of the physiological and pharmacological activities of BRPs from amphibian skin secretions are needed in the future.
